# The Effect of Environmental Disasters on Endocrine Status, Hematology Parameters, Body Composition, and Physical Performance in Young Soccer Players: A Case Study of the Aral Sea Region

**DOI:** 10.3390/life13071503

**Published:** 2023-07-03

**Authors:** Valerii O. Erkudov, Kenjabek U. Rozumbetov, Francisco Tomás González-Fernández, Andrey P. Pugovkin, Ilal I. Nazhimov, Azat T. Matchanov, Halil İbrahim Ceylan

**Affiliations:** 1 Department of Normal Physiology, St. Petersburg State Pediatric Medical University, 194100 Saint Petersburg, Russia; verkudov@gmail.com; 2 Department of General Biology and Physiology, Faculty of Biology, Karakalpak State University, Nukus 230100, Uzbekistan; rozumbetov96@mail.ru (K.U.R.); ilal.najimov@gmail.com (I.I.N.); matchanovazat@gmail.com (A.T.M.); 3 Department of Physical Education and Sports, Faculty of Sport Sciences, University of Granada, 18071 Granada, Spain; ftgonzalez@ugr.es; 4 Department of Biotechnical Systems, Faculty of Information Measurement and Biotechnical Systems, Saint Petersburg Electrotechnical University «LETI», 197022 Saint Petersburg, Russia; 5 Physical Education and Sports Teaching Department, Kazim Karabekir Faculty of Education, Ataturk University, Erzurum 25240, Turkey

**Keywords:** testosterone, IGF-1, somatotype, youth, soccer

## Abstract

The Aral Sea region (Uzbekistan) is infamous because of the ecological disaster characterized by the disappearance of the Aral Sea due to excessive uncontrolled water intake for agriculture needs. A new desert occurrence, soil and climate aridization led to pesticide and toxic metals environment pollution. The impact of environmental conditions in some areas of Uzbekistan on the health of soccer players is not as noticeable as, for example, the effectiveness of training, so it is not widely discussed in scientific papers. The aim of the present study was to study the features of endocrine status, hematology parameters (e.g., red blood cells (RBC) and hemoglobin (Hb)), and their influence on body composition and physical fitness performance in local young soccer players of the Aral Sea region as the territory of ecological disaster. The study involved 60 male soccer players aged from 18 to 22 years. Participants were divided into two groups: the experimental group (EG), which consisted of 30 soccer players living on the territory of the Aral ecological disaster region, and the control group (CG), which included 30 soccer players, natives of the ecologically favorable region of Uzbekistan. All volunteers had anthropometric measurements, concentrations of insulin-like growth factor-1 (IGF-1), total testosterone (TT), estradiol (E2), cortisol (C), RBC, and Hb count. Moreover, Yo-Yo Intermittent Recovery Test Level 1 (YYIRT1) and professional skills tests such as dribbling shuttle test (DSt) and goal accuracy test (GAt) were assessed. When comparing the CG group to the EG group, it was observed that the EG group exhibited statistically significantly reduced levels of TT and E2 (*p* < 0.05). No significant statistical difference was observed between the two groups in terms of IGF-1 and C (*p* > 0.05). Regarding hematological parameters, Hb, Ht, and mean corpuscular volume (MCV) were found to be significantly lower in the EG compared with the CG (*p* < 0.05). Moreover, the distance covered in the YYIR1 test was found to be significantly lower in soccer players within the EG compared with the CG (*p* < 0.05). Additionally, it was determined that there was no significant difference between the groups in terms of DSt and GAt values (*p* > 0.05). Lastly, significant differences were observed between the EG and CG in terms of anthropometric characteristics (diameters, skinfold, and somatotype profile) (*p* < 0.05). The present study showed that the changes in evaluated characteristics might result from the complex influence of endocrine-disrupting chemicals, the content of which is high in the environment of the Aral Sea region. The results obtained may help monitor the health of athletes living in an environmentally unfriendly environment.

## 1. Introduction

Soccer is one of the most popular games in the world [1]. In Uzbekistan, sports people go from non-professionals who play for recreational purposes to elite athletes, having contracts with the best clubs of the republic and worldwide. To this end, coaches, scouts, and sports medicine professionals often select the most likely to succeed athletes based on their experience and subjective criteria [2]. The chosen soccer players are maturated [3,4,5], have high aerobic endurance, and technical skills and fitness parameters (strength, speed, flexibility, coordination, etc.) [6]. Thus, physiological and anthropometric criteria of sports selection are also used to identify sports talent [7,8,9].

The impact of environmental conditions in some areas of Uzbekistan on the health of soccer players is not as noticeable as, for example, the effectiveness of training, so it is not widely discussed in scientific papers. The southwestern territory of Uzbekistan, named the Aral Sea region, is infamous because of the ecological disaster that has existed there since the middle of the 20th century [10]. Excessive uncontrolled water intake from the Amudarya River for agriculture needs led to disruption of the natural migration of groundwater [11]. This was the cause of the disappearance of the Aral Sea, creating a new desert known as the Aralkum, soil and climate aridization, and the occurrence of salt storms [11,12].

The Aral ecological disaster has at least two complementary effects on the health of athletes: air pollution by microparticles and pollutants [13,14] as well as the accumulation of heavy metals [15,16] and organochlorine pesticides (OCPs) [15,17,18,19]. Regarding this, a previous study indicated that significant differences in blood levels were found for four analyzed OCPs in subject from north vs. south parts of Aral see regions, namely lindane (1.8 ± 0.02 mg/dL vs. 0.4 ± 0.04 mg/dL), diendrin (16.9 ± 0.31 mg/dL vs. 3.1 ± 0.37 mg/dL), dichlorodiphenyltrichloroethane (DDT) (17.8 ± 0.27 mg/dL vs. 10.9 ± 0.26 mg/dL), and endrin (3.8 ± 0.09 mg/dL vs. 0.48 ±0.07 mg/dL) [19]. Several studies revealed changes in physiological parameters relative to air pollution and exercise. For instance, Boussetta et al. [13,18] and Zacharko et al. [14] found endurance performance, hematological parameters, and hemodynamics and respiratory reactivity were significantly affected by pollution. OCPs [19] and heavy metals [16] accumulate in the Aral Sea region, and enter the human organism with food and water, where they have an endocrine-disrupting chemicals (EDC) effect [15,17,20,21]. EDCs are known for their negative effect on the biosynthesis and metabolism of testosterone [15,21,22,23], estrogens [21,24], and insulin-like growth factor 1 [20,25,26]. A previous study showed the relationship between body structure and possible anti-androgen and antiestrogen effects of EDCs athletes [27] and non-athletic young men [28] living in the Aral Sea region. Due to prolonged intensive workloads, soccer players have been reported to have high testosterone [29] and IGF-1 [3,30], which are associated with better game performance, anthropometric and physiological characteristics, and a high probability of selection to professional teams [3,30,31].

Taking into account previous research, which has found low levels of red blood cells (RBCs) and hemoglobin (Hb) in individuals with high plasma concentrations of organochlorine pesticides (OCPs) in the Aral Sea region [32,33], this study aimed to investigate the relationship between endocrine status, body physique, and physical fitness performance in young soccer players. The Aral Sea region is known for its high concentration of environmental pollutants due to ecological disasters. Hence, we hypothesized that exposure to such negative environmental factors can potentially affect the oxygen-carrying capacity of blood and subsequently alter physical performance levels in young soccer players.

Given the potential for environmental pollutants to impact the oxygen-carrying capacity of blood, this study aimed to shed light on the impact of ecological disasters on young soccer players’ physical health and performance. By studying the effects of endocrine status and hematology parameters on body physique and physical fitness, this research may help identify ways to monitor and mitigate the health risks posed by negative environmental factors in the Aral Sea region.

## 2. Materials and Methods

### 2.1. Study Design

The participants were divided into two groups: The experimental group (EG) consisted of 30 soccer players, grown and since birth living on the territory of the autonomous Republic of Karakalpakstan (Uzbekistan) in the region of the Aral ecological disaster. The control group (CG) included 30 soccer players, natives of Khwarazm region of Uzbekistan

In total, players from both groups trained for 216 h over 52 weeks in 2021/2022 from November to October. The entire cycle consisted of three periods: a «training period» that included only training (24.6 weeks from November 2021 to late April 2022), a «playing period» that had training and regular season amateur college team games (23.4 weeks from late April to early October), and a vacation (4 weeks from October to the beginning of the next cycle). All tests were performed at the end of April 2022 from weeks 23 through 25 of the training cycle.

### 2.2. Participants and Study Area

A total of 60 male soccer players aged from 18 to 22 years were recruited to participate in our study. Of them, 30 athletes were from a team based at Karakalpak State University named Berdakh (Nukus, Uzbekistan), and another 30 participants trained on Urgench state university (Urgench, Uzbekistan) team. The two teams, in equal proportions, comprised four goalkeepers, 20 defenders, 20 central midfielders, eight wingers, and eight attackers. The minimum sample size for our study was determined using G-power software version 3.1.9.7 (University of Dusseldorf, Dusseldorf, Germany). In accordance with our study’s design, an a priori analysis utilizing t tests was conducted to calculate the statistical power. Specifically, we employed the Wilcoxon–Mann–Whitney test (two groups) with the following parameters: α error probability (α err prob) = 0.05, power (1-β error probability) = 0.80, allocation ratio of 1, and effect size of 0.70 [14,15,18]. Based on the analysis, it was determined that a total of 56 participants (with 28 participants in each group) would be required to achieve 81.07% of the desired power.

Participants started training at the age of 10–12 years. The training program consisted of three weekly workouts (4,8 h/week, 18 h/month, 216 h/year). All thirty students in the soccer departments of both universities volunteered for this study because the physical fitness assessment is an additional selection factor for professional teams. None of them reported any sports injuries, using any supplement that could affect growth and maturation, iron supplementation, or donated blood at the time of the investigation. The players followed the same nutritional and hydration protocol as during the soccer season. In all tests in this study, athletes participating in 80% of competitions and training for the whole season could not perform additional exercise/training regimens.

The study was conducted according to the guidelines of the Declaration of Helsinki and was approved by the local ethics committee of the St. Petersburg State Pediatric Medical University (protocol No. 17/3, 10 May 2018). Informed consent was obtained from all individual participants included in the study.

**Figure 1:**
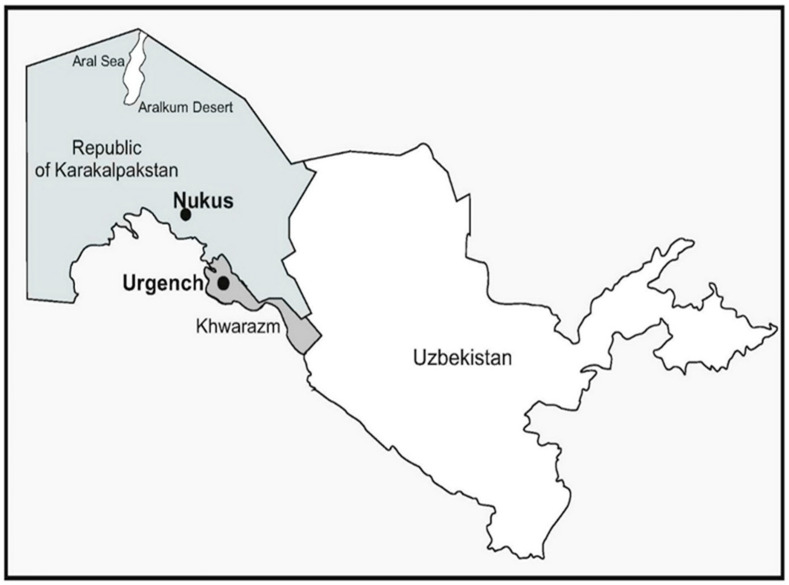
Interposition of Nukus (EG) and Urgench (CG) in Uzbekistan (Central Asia).

These groups belong to two similar regions of Uzbekistan: autonomous Republic of Karakalpakstan and Khwarazm region (see Figure 1, for more information). The territory of Republic of Karakalpakstan has the largest area in the Aral Sea region (16 MHa approximately) and developing ecological disaster. In the Khwarazm region, there is practically no aridization, salinization, and soil and water pollution by pesticides and heavy metals due to the influence of the Khwarazm oasis [11]. The air does not contain large amounts of pollutants and almost no dust and salt storms [11]. Drying lakes can be very effective sources of wind-borne atmospheric dust. Winds over the Aralkum vary from year to year, so modeling predicts that most of the Aralkum dust is transported eastward during the year. As storm winds sweep across these areas, dense dust containing pollutants is deposited over vast remote areas and is also inhaled by people and animals. In addition, polluted river water used for irrigation contaminates the soil with toxic chemicals, which are then absorbed by vegetables and other foodstuffs [11]. Special studies have reported higher concentrations of heavy metals in soil samples from the station located at Takhiatash, suburban of Nukus city (up to 300 ppb), compared with samples from Urgench station (up to 198 ppb) [11]. For more information about both regions, see http://www.cawater-info.net/index_e.htm and https://visibleearth.nasa.gov/images/41377/aral-sea/41378l (accessed on 20 February 2023) (Figure 1).

### 2.3. Procedures

During the study, participants attended a single outpatient visit where their medical history was recorded, and they underwent a comprehensive physical examination. Throughout the three-day study period, participants were instructed to refrain from any dietary disruptions, maintain proper hydration, avoid stress, and refrain from engaging in excessive physical activity. To reduce measurement variations, the same experienced investigator conducted all the evaluations. Physical fitness characteristics assessments were supervised by the main researcher and physical coach responsible for these athletes, considering the capabilities that they worked on for each match. We followed the guidelines of the American College of Sports Medicine [34] to ensure the safety of the participants.

On the first day of the experiment, two experienced investigators performed anthropometric measurements in the morning at a comfortable temperature (temperature:18–20°C; humidity: 50–60%) in the isolated, artificially illuminated medical room. Participants were dressed in light clothing. On the second day, the participants first warmed up for 15 min. Subsequently, they performed the Goal Accuracy Test and Dribble Shuttle Test (three times, with a rest period of 3 min between each). Moreover, there was a 3 min rest period between the two tests. Lastly, the participants were subjected to the Yo-Yo Intermittent Recovery Test Level 1 (YYIRT1). The measurement session was completed after the participants performed cool-down exercises following all the tests. All physical fitness characteristics evaluations were performed at approximately the same time of the day (between 4:00 and 5:00 pm) and in similar environmental conditions (temperature:18–20°C; humidity: 50–60%) [34] on the grass or in the gym. Moreover, twenty-four hours after the last training, the players were invited to a private medical clinical and biochemical laboratory, where blood samples were taken by an authorized medical professional.

### 2.4. Data Collections Tools

#### 2.4.1. Anthropometric Measures Assessment

The body physique parameters were measured according to the method proposed earlier [35,36,37]. To measure standing height, participants stood without shoes and socks with their lower back as close to the stadiometer as possible, until the back of the head, shoulder blades, buttocks, and heel touched the stadiometer, and their legs were placed together. Then height was measured by the rod from above the head. To measure sitting height, the participant was instructed to sit on a 50 cm height box, facing forward. They were asked to position their lower back as close as possible to the stadiometer, ensuring that their pelvis was in contact with the stadiometer. If feasible, they were also encouraged to have their shoulders touching the stadiometer. They kept the upper body as straight and flat as possible and put their hands on their feet. Then the height was measured between the highest point of the head (vertex) and the support plan of the hip (Ischial spines) where they were sitting on a box. They kept their head in the Frankfurt horizontal plane.

Measurement of 23 parameters, including body mass, body mass index (BMI), stature, sitting height, circumference of limbs, leg length, size of chest, pelvis, large joints diameters, and skinfold thickness were performed on all participants. The stature and sitting height were assessed using mobile stadiometer SECA Model 217, Germany. Participants were unshod to measure the standing height (stature), with the heels, hips, shoulder blades, and back of the head as close as possible to the stadiometer, with feet placed beside each other. For sitting height, participants sat on a 50 cm bench and were instructed to bring their buttocks as close as possible to the stadiometer, holding their upper body straight and placing their hands on their feet, at which point, their height was assessed. The distance between the highest point of the head and the bench was calculated as sitting height. Body mass was measured with electronic medical scales SECA Model 217, Germany, to the nearest 100 g. BMI was calculated using the Quetelet formula. The leg length was calculated as stature minus sitting height. The anterior-posterior (sagittal) and biacromial chest diameters (BACDs), as well as pelvic biiliac diameter, were measured using a large spreading caliper (KAFA, Moscow, Russia). The wrist, knee, elbow, and ankle diameters were measured by sliding caliper (KAFA, Moscow, Russia), measurement accuracy for both calipers was up to 0.1 cm. Non-elastic tape SECA Model 200, Germany, was used to take limb circumferences with 0.01 accuracy. The skinfold thickness was assessed using a professional caliper ET MEASURE model SK-101, China, with spring calibrated to produce equal pressure (0.01 kg/mm2) on both sides of the fold, and the accuracy of the measurement was 0.2 mm.

#### 2.4.2. Somatotype Assessment

The somatotype was assessed by the Heath–Carter method. Body somatotype the three-dimensional distance from a profile to the mean of all profiles (endomorph, mesomorph, and ectomorph) and height-to-weight ratio (HWR), according to Carter and Heath [37,38]. Numerical values of body mass, stature, knee and elbow diameters, upper arm and calf circumferences, triceps, subscapular, and suprailiac skinfolds were substituted into the calculation formulas of ecto-, meso-, and endomorph components of somatotype [38].

#### 2.4.3. Physical Fitness Parameters

##### Yo-Yo Intermittent Recovery Test Level 1 (YYIRT1)

The endurance capacity of the players was measured by the YYIRT1 test [39]. Players repeated 20 m shuttle runs back and forth between the starting and finish lines marked by cones, at progressively increasing speeds dictated by an audio beep emitted from a CD player. Between each shuttle, players had a 10 s period of walking around a cone placed 5 m from the starting line. Failure to complete the shuttle run on two successive occasions resulted in the termination of the test and the distance covered represented the final test result. The club’s coaches habitually conducted the YYIRT1 during the season for all age-categories, and the test leaders were also highly familiar with all testing procedures [40,41].

The dribbling shuttle test and goal accuracy test assessed the professional performance skills used to control the examination included in the sport selection procedure for professional soccer teams in Uzbekistan.

##### Dribbling Shuttle Test (DSt)

**Figure 2:**
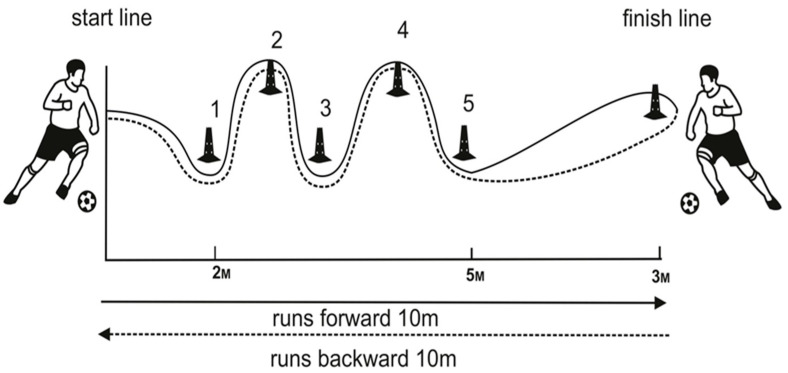
The DSt procedure.

The DSt procedure is sketched and presented in Figure 2. Football cones were placed by the scheme. The volunteer started moving from the «start line». It was necessary to run at a maximum speed of 20 m (forward and backwards), while dribbling the ball. While running along in a spiral, going around the cones from 1 to 5, the player reached the «finish cone». After that, the subject moved back, spiraling around the cones from 5 to 1, reaching the «start line». The procedure was recorded on a professional video camera. The video was viewed in slow motion, noting two parameters: the number of touches of the ball and the time it took to complete the test.

##### Goal Accuracy Test (GAt)

The player needs to hit precisely the central zone (zero quadrants) of empty football goals of standard sizes from a distance of 30 m. As a result, the number of successful hits from 5 attempts was recorded.

#### 2.4.4. Blood Sample Test

Venous blood samples were taken between 7:00 and 8:30 a.m. following an overnight fast. Blood samples were centrifuged at 1500 rpm for 10 min to obtain the serum. Hormonal (insulin-like growth factor 1 (IGF-1), total testosterone (TT), estradiol (E2), and cortisol ©) and hematological parameters (Red blood cells (RBC), Hemoglobin (Hb), hematocrit (Ht), mean corpuscular hemoglobin (MCH), mean corpuscular hemoglobin concentration (MCH), mean corpuscular volume (MVC), and iron concentrations (Fe)) were registered. In this sense, IGF-1, TT, E2, and C concentrations in serum were measured by the direct solid-phase chemiluminescent enzyme immunoassay («sandwich» method) with commercial test kits using MR-96A Mindray microplate reader, Shenzhen Mindray Bio-Medical Electronics Co., Ltd., China. Regarding hematological parameters, in addition, RBC, Hb, Ht, MCH, and MCV were determined on a Mindray BC-20s hematology analyzer, Shenzhen Mindray Bio-Medical Electronics Co., Ltd., China. In addition, Serum Fe concentrations were measured by calorimetry using semi-auto chemistry analyzer BA-88A Mindray, Shenzhen Mindray Bio-Medical Electronics Co., Ltd., Shenzhen, China. The inter-assay coefficient of variations and sensitivity were: 4.3–7.8% and 0.15 ng/mL for IGF-1; 4.4–9.3% and 0.33 nmol/L for TT; 6.1–8.5% and 6.0 pg/mL for E2; 3.8–8.1% and 8.7 nmol/L for C; and 4.5–9.2% and 2.0 µmol/L for Fe.

#### 2.4.5. Statistical Analysis

A comparison of anthropometric, hematological parameters, hormonal, and Fe concentrations, distance in YYIR1, DSt time, and numbers of ball touching between participants in the EG and CG was conducted using the Mann–Whitney U-criterion. The decision to use a non-parametric test was made after examining the data using the Shapiro–Wilk test, which indicated a deviation from normal distribution. Categorical variables, the numerical ratio of the distribution effective shots on GAt in the EG and CG, were analyzed using Fisher’s exact test for feature conjugation tables 5 × 2.

Where the samples analyzed had statistically significant differences, a standardized effect size Cohen’s d was calculated using standard formula [42]. The Cohen’s d was interpreted according to the method suggested by [42]: 0–0.2, negligible effect; 0.2–0.5, small effect; 0.5–0,8, moderate effect; more than 0.8, high effect.

The calculations were performed using the statistical data processing software Past version 2.17, Norway, Oslo, 2012, StatXact-8 statistical data processing algorithm with Cytel Studio software package version 8.0.0 and SPSS software (version 20.0; SPSS, Inc., Chicago, IL, USA). An Excel application from Microsoft Office 2010 Exploratory Software for Confidence Intervals (ESCI-JSMS), Melbourne, Australia, 2001, was used to calculate Cohen’s d. The results were considered to be significant at *p* < 0,05. All continuous data are presented as arithmetic mean (µ) and 95% confidence intervals (CI). Categorical data were presented as fractions with 95% CI.

## 3. Results

As shown in Table 1, a statistically significant difference was observed in the hormonal profiles of the EG and CG groups when compared (*p* < 0.05). According to this, compared with the CG group, the EG group was found to have significantly lower levels of TT (*p* = 0.001, effect size: 1.02, large effect) and E2 (*p* = 0.001, effect size: 0.89, large effect). No statistically significant difference was found between the two groups regarding IGF-1 and C (*p* > 0.05).

Regarding the hematological parameters, there was a statistically significant difference in RBC (*p* = 0.002, effect size: 0.88, large effect), Hb (*p* ≤ 0.001, effect size: 1.31, large effect), Ht (*p* = 0.001, effect size: 1.52, large effect), and MCV (*p* = 0.02, effect size: 0.67, moderate effect) values between the two groups. These parameters were found to be significantly lower in the EG compared with the CG. Moreover, no significant differences were found between the EG and CG groups in other hematological parameters such as Fe and MCH (*p* > 0.05). Additionally, the distance in the YYIR1 test (*p* = 0.04, effect size: 0.46, small effect) was statistically significantly lower in soccer players in EG compared with CG. Finally, it was observed that the values related to DSt (number of ball touching, and DSt time) did not differ significantly between the groups at a statistically significant level (*p* > 0.05) (Table 1).

As shown in Table 2, the distribution of effective shots was found to be homogeneous in GAt, and there was no significant difference between the participants in the EG and CG groups (*p* > 0.05). Based on these results, it can be concluded that the presence of effective shots is independent of the region of residence of study participants.

**Figure 3:**
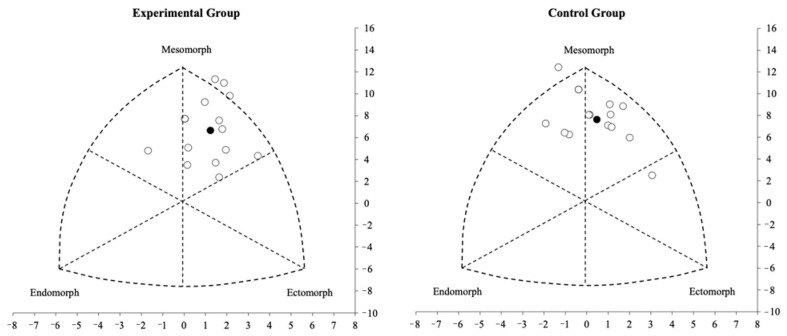
Somatotype profile distribution of young soccer players. The white circles are the individual somatotypes and the black circle is the mean profile.

When Table 3 is examined, it can be seen that the sitting height (*p* = 0.01, effect size: 0.64, moderate effect), body weight (*p* = 0.04, effect size: 0.55, moderate effect), BACD (*p* = 0.02, effect size: 0.69, moderate effect), APCD (*p* =0.02, effect size: 0.71, moderate effect), PBD (*p* = 0.01, effect size: 0.71, moderate effect), wrist diameter (*p* = 0.01, effect size: 0.53, moderate effect), knee diameter (*p* = 0.001, effect size: 0.83, large effect), elbow diameter (*p* = 0.001, effect size: 0.82, large effect), ankle diameter (*p* = 0.018, effect size: 0.66, moderate effect), suprailiac skinfold (*p* = 0.04, effect size: 0.34, small effect), and calf back skinfold (*p* = 0.02, effect size: 0.60, moderate effect) of the CG were significantly higher than those of the EG. Moreover, only leg length was found to be significantly higher in the CG group compared with the EG group in terms of anthropometric characteristics (*p* = 0.01, effect size: 0.64, moderate effect). Lastly, there were significant differences in ectomorph (*p* = 0.01, effect size: 0.76, moderate effect) and mesomorph somatotype component (*p* = 0.02, effect size: 0.61, moderate effect) between EG and CG groups. Additionally, it was observed that soccer players in the EG predominantly exhibited mesomorph somatotype component (medium effect size) and ectomorph somatotype component compared with the CG group (Figure 3).

## 4. Discussion

The scientific literature has predominantly focused on the impact of air pollution on the physical condition of athletes, including effects on aerobic performance, hematological parameters, and strength characteristics of soccer players [13,14,18,43,44,45,46]. The present study represents the first to characterize the features of the anthropometric profile, endurance performance, and professional skills in soccer players who grew up and lived in the environmentally unfavorable Aral disaster region. The limited research on this topic highlights the need for further investigation. The present study provides a valuable contribution to this field by characterizing the features of an anthropometric profile, endurance performance, and professional skills in soccer players affected by ecological disasters. The results of our study may help inform strategies to monitor and mitigate the health risks associated with negative environmental factors, particularly in regions with a history of ecological disasters, such as the Aral Sea.

In our study, we sought to observe a homogeneous group of soccer players, considering their gender, age, level of training, experience, training schedule, and level of fatigue. We found no high plasma cortisol concentrations or differences in participants from either group. Increased cortisol concentration is a valid marker of overtraining in athletes [47,48,49]. This result indicates the absence of overtraining and fatigue in the volunteers in this study. It should also be noted that TT [48,49,50,51], E2 [52], RBC, Hb, Ht [48], and YYIRT-1 [48] were comparable to the values of these parameters in soccer players published in other papers.

Prolonged physical activity increases IGF-1 [3,30] and testosterone [29,52]. IGF-1 positively correlates with athletes’ strength and performance [53] results in jump tests [3,40]. These hormones increase muscle mass through protein synthesis, especially in fast muscle fibers, leading to increased muscle activity [52]. Testosterone suppresses aseptic inflammation that develops during muscle exercise [35,36,52], and positively affects overall performance and recovery after exercise [52,54]. We did not find any differences in professional performance skills between EG and CG athletes. The anti-androgenic effect of the environment on the performance of athletes who grew up in the epicenter of the Aral Sea environmental disaster was insufficient. Moreover, we do not assume the effect of reduced concentration of sex steroids on the physical performance of soccer players.

Soccer is a rigorous sport in which the players must exercise and perform at greatly uneven intensities distributed over time in a highly discontinuous manner [50,52], YYIRT1 was developed by J. Bangsbo as a means of assessing a soccer player’s endurance performance under conditions simulating high-intensity intermittent exercise which requires players aerobic and anaerobic competence [39]. It is one of the main tests to evaluate soccer player’s aerobic capacity of with high reliability and specificity [55] and was successfully applied in the study [3,4,13,56]. The present study revealed lower endurance performance in EG athletes when performing YYIRT1. It is known that YYIRT1 performance is related to the sufficiency of both aerobic and anaerobic metabolism [57,58]. Furthermore, research suggests that the aerobic energy pathway plays a significant role in endurance performance, and a decrease in this pathway may be linked to a reduction in the oxygen-carrying capacity due to Hb deficiency, and a lower number of RBC [56,59]

Participants from EG compared with players from the CG had a decreased amount of RBC and Hb, combined with normochromia, equal plasma iron content, and a tendency to microcytosis. Thus, they are assumed to have no impairment of hemoglobin synthesis, the efficiency of which depends on iron intake, all other things being equal. Less total hemoglobin in them is transported in fewer small erythrocytes, so the saturation of each erythrocyte is equivalent. The value of total Hb in participants in EG depends on the number of RBC, that is, on erythropoiesis, but not on the Hb saturation of each erythrocyte. If the number of RBCs in athletes from both groups were not different, with equal MCH we would obtained the same Hb content in them. It is known that Hb, RBC, Ht, MCV, and MCH do not change in soccer players during the competitive season [56].

Currently, the mechanisms of the effects of pesticides and pollutants on hematopoiesis are not fully understood. However, analysis of the literature suggests a direct and sex steroid-mediated pathway of reduced RBC production under the action of OCPs [32,33]. Low RBC and Hb levels combined with high plasma OCPs content have been found in residents of environmentally unfriendly regions contaminated with these pesticides [32,33]. Special studies have shown that OCPs are toxic to bone marrow [60]. In addition, OCPs-mediated modification of the cell cycle has also been found to be a likely mechanism of hematopoietic toxicity and aplasia of hematopoietic stem cells and their immediate progeny [61].

Testosterone normally promotes erythropoiesis by direct effects on the hematopoietic stem cell [62,63] or by permissive interaction with erythropoietin [64] and IGF-1 [65]. Similar mechanisms were found in estrogen [66]. In athletes, plasma androgen concentrations correlate with YYIRT1, Hb, and RBC [50,56]. Thus, the suppression of proliferative activity of hematopoietic stem cells may be a consequence of the endocrine-depleting effect of OCPs on sex steroids [21,22].

Decreased RBC in peripheral blood may be a consequence of damage to hematopoietic cells and mature erythrocytes because oxidative stress increases lipid peroxidation [67]. Intense muscle exercise in athletes leads to microdamage of muscles, lipids, and oxidative stress [67], which are increased by exposure to pollutants such as carbon monoxide, nitrogen dioxide, pollutants such as ozone, and others [44,67]. Similar effects have been found in OCPc [68]. Inhalation of particulate matter and gaseous pollutants can cause acute autonomic nervous system imbalance, systemic pro-inflammatory reactions, and endothelial dysfunction vasoconstriction [18]. Athletes [43,44] and soccer players in particular [45] appear to be more vulnerable to high concentrations of atmospheric air pollutants. This fact is attributed to an increase in inhaled pollutants with increased minute ventilation during exercise, which causes an increased proportion of air inhaled through the mouth, absorption of pollutants, and distribution in the body [14,69]. Alveolar air pollution leads to redistribution of oxygen, decreased diffusion, and hemoglobin saturation [69]. Athletes who train in areas with increased air pollution have been found to have decreased YYIRT1 performance, Hb level, and partial oxygen tension in the blood [13,14,18,43,44,45].

It should be noted that the effect size of these changes in athletes from the environmentally disadvantaged region was not high. The distance covered when performing YYIRT1 correlates not only with hematological parameters but also the efficiency of the test is determined by the compensatory reactions of blood circulation and respiration, which develop in trained athletes [56]. Increased erythropoiesis may occur due to an androgen-mediated increase in muscle mass and be the cause of partial compensation of carry oxygen blood capacity [63].

Thus, the presumed endocrine-disrupting and hematopoiesis-reducing effects of unfavorable environmental factors were not critical for the changes in the physical fitness characteristics of the examined soccer players. We observed the most pronounced effect of the possible anti-androgenic and anti-estrogenic effects of long-term persistent OCPs on volunteers’ body structure and shape.

It is known that the formation of an anthropometric profile in soccer players is a consequence of the influence of two interrelated components: targeted selection of young people for sports [3,4,7,40], and prolonged physical activity during critical growth periods [9,70]. Athletic selection most effectively achieves its goals among pre-pubertal children and adolescents [9,70]. Coaches strive to recruit the most mature players for their age who want to become professional players in the future [3,4]. In Uzbekistan, the selection is based on the same anthropometric criteria for all soccer players in different regions. Athletic training during puberty has positive effects on growth [9,50,70], stimulating testosterone synthesis and release [52,71] and IGF-1 [4,30].

Sitting height is a convenient and valid anthropometric parameter for assessing child maturity in pre-pubertal and early pubertal life [4,40,49,72]. It is believed that the greater the sitting height and shorter the leg length, the less time there is before the peak growth rate at a pubertal spurt [73]. Maturational performance is positively correlated with growth hormone concentrations [4], IGF-1 [4], and testosterone [29]. It is associated with speed and strength motor qualities and soccer technical skills [6] as well as YYIRT1 [5,6,7,40].

Soccer players are characterized by a mesomorph body type [72,74] with a low percentage of fat and developed muscles [5,74]. They may also show high ectomorph values [5,51], and signs of late maturation [72]. The predominance of muscle mass and a deficit of fat mass can provide effective control and body weight lifting during running of varying intensities, jumping, turning, kicking the ball, and dribbling [5]. Mesomorph is a predominant factor in injury reduction [5].

The data obtained in work indicate a delay of maturation in the pre-pubertal period and in puberty in soccer players living in the conditions of the Aral ecological disaster. The anthropometric features revealed may be a consequence of the endocrine-disrupting effects of OCPs. We obtained lower TT and E2 values in soccer players from EG compared with CG volunteers with equal IGF-1 concentration. OCPs are known to decrease the rate of maturation due to anti-androgenic [15,21,22,23] and anti-estrogenic [21,24] actions. OCPs have also been reported to show anti-IGF-1 effects, which were not detected in this paper. During puberty, there is a permissive interaction between sex steroids and IGF-1, which provides limb elongation due to increased sensitivity of the growth plate [75,76,77] shoulder, forearm, lower leg, and thigh to these hormones [78]. The anti-androgenic and anti-estrogenic effects of OCPs lead to a decrease in the growth rate of leg and arm bones, with little or no stagnation in thoracic growth [15,21,22,23]. All the above may change the anthropometric profile of the selected at puberty based on the level of maturity of soccer players who grew up in environmentally disadvantaged conditions. Players acquire ectomorph characteristics: low sitting height to leg length, flattened chest, narrow pelvis, smaller skinfold thickness, thin bones, increased ectomorph values, and decreased mesomorph component of Heath–Carter’s somatotype.

Large joint diameters are used as an anthropometric marker of bone mass [79]. Increased physical activity [80] and accelerated maturation during puberty increase bone mass [4,81]. Testosterone [81], estrogen [81,82], and IGF-1 [81] levels correlate with bone mass and density in athletes. Testosterone [81] and estrogen [82] are necessary for bone mineralization. Androgens also increase intracellular calcium transport [52] and have a permissive interaction with growth hormone and calcitonin for osteoblast stimulation [82]. Some researchers have reported that increased body weight, due to increased bone and muscle mass, is positively correlated with impact force in soccer players [80]. The anti-androgenic effect of OCPs was probably the reason for the decrease in wrist, knee, elbow and ankle diameters and body weight in volunteers from EG compared with the soccer players from the CG. These anthropometric features may be the reason for the change in impact force in athletes living in the region of environmental disaster, which may be the subject of further research.

Despite the significant findings and potential implications, several limitations to this study should be considered. Firstly, the study only included male soccer players; therefore, the results may not be generalizable to female athletes or athletes in other sports. Additionally, the sample size was relatively small, which may limit the study’s statistical power and reduce the findings’ representativeness.

Secondly, the study was cross-sectional, meaning it only provided a snapshot of the athletes’ health status and physical performance at a single time. As such, it is not easy to conclude causality or the long-term effects of environmental factors on the health and performance of athletes. Thirdly, the study only assessed a few ecological factors and did not consider potential confounding variables that could influence the results. For example, the athletes’ dietary habits, lifestyle, and other environmental exposures were not measured, which may have affected the study’s findings.

Finally, the study did not investigate the potential mechanisms underlying the observed effects of environmental factors on the athletes’ health and performance. Further research is needed to identify the specific chemicals or toxins responsible for the observed effects and elucidate the biological pathways through which they act.

In fact, for a depth analysis of the factors that concurred for justifying the changes, it would be necessary to describe and quantify the training process and load during the period and use this as a moderator. We consider this a bias, and the generalization of the findings should be avoided. Future studies should consider monitoring the training load and use this sum of training exposure as a possible co-variable. Despite that, both groups competed in the same category, and age, stature, body mass, and body mass index were controlled to observe the previous difference. It is also expected that the training process for these categories can be relatively similar without major differences in the overall load imposed or advanced training methods. Moreover, our study did not consider exposure time to a specific training goal. This means that in some cases, higher exposure to cardiorespiratory training may have more impact in one group than in another. Thus, future studies should consider exposure time to a given training method as a possible co-variable.

In addition, future research can include an observational study in which actions involving decision making are obtained and the normal behavior of the footballer is evaluated, such as vertical and horizontal passes, comparing by context, as well as increasing the information on the fluctuation of physical conditioning during the season, when the air quality is good, and after several weeks/months, when this quality is not favorable. This study is one of the pioneering investigations conducted in Spain concerning this particular issue and focused on youth football players. This type of study may help in the improvement and control of the physical–biological performance of football players in training stages, especially from the sports medicine perspective.

## 5. Conclusions

The present study objectively characterizes the physical development and performance of local young soccer players in the Aral Sea ecological disaster region, focusing on their anthropometric profile, hormonal regulation, and oxygen-carrying capacity. The findings reveal that these athletes display ectomorph somatotype features, including a low sitting height, flattened chest, narrow pelvis, smaller skinfold thickness, thin bones, and an increased ectomorph and decreased mesomorph component of Heath–Carter somatotype. It was suggested that these physical development features may be influenced by environmental endocrine-disrupting substances that reduce the content of sex steroids in athletes from the observation group. Furthermore, the negative environmental factors in the Aral Sea region are likely responsible for the soccer players’ decreased levels of RBC and Hb. This decline may have reduced oxygen-carrying capacity and decreased endurance performance during the YYIRT1 test. However, while hormonal changes, blood cell count, and somatotype features were affected, there was no significant influence on the professional skills of the examined soccer players.

These findings are significant as they provide insights into the impact of environmental factors on athletes’ physical development and performance. This information can be valuable for monitoring the health status of athletes living in regions with ecological issues. The results of this study also contribute to the limited literature on this topic, and further research is needed to explore the complex relationships between environmental factors, physical development, and athletic performance.

## Figures and Tables

**Table 1:** Hormonal profile, hematological parameters, and physical fitness characteristics in soccer players from EG and CG (µ; 95%CI).

Parameter	EG (Mean, 95% CI)	CG (Mean, 95% CI)	*p*-Value	Cohen’s d
Hormonal parameters				
IGF-1 (ng/mL)	441.37 (415.90; 466.83)	429.83 (406.21; 453.46)	0.52	–
TT (nmol/L)	27.65 (26.51; 28.79)	32.69 (30.35; 35.04)	0.001 **	1.02
E2 (pg/mL)	20.85 (18.45; 23.24)	26.87 (24.20; 29.54)	0.001 **	0.89
C (nmol/L)	237.88 (206.93; 268.82)	232.69 (203.34; 262.05)	0.97	–
Hematological parameters				
RBC × 10^12^/L	4.67 (4.46; 4.88)	5.13 (4.95; 5.31)	0.002 **	0.88
Hb (g/L)	138.27 (136.52; 140.01)	154.57 (143.22; 147.92)	0.001 **	1.31
Ht (%)	40.40 (39.07; 41.23)	46.40 (44.29; 48.01)	0.001 **	1.52
MCH (pg)	29.92 (28.76; 31.17)	28.53 (27.83; 28.24)	0.12	–
MCV (fl)	86.07 (84.64; 89.09)	90.57 (88.65; 92.49)	0.02 *	0.67
Fe (µmol/L)	16.98 (15.77; 18.19)	17.68 (16.35; 19.01)	0.42	–
Physical Fitness parameters				
YYIR1 (m)	1528.00 (1343.20; 1713.17)	1742.67 (1572.67; 1912.60)	0.04 *	0.45
DSt (nbt)	38.00 (36.00; 40.00)	39.00 (38.00; 42.00)	0.12	–
DSt (sec)	26.63 (25.37; 27.90)	27.51 (26.42; 28.59)	0.36	–

Note: EG: experimental group; CG: control group; CI: confidence interval; IGF-1: insulin-like growth factor 1; TT: total testosterone; E2: estradiol; C: cortisol; RBC: red blood cells; Hb: hemoglobin; Ht: hematocrit; MCH: mean corpuscular hemoglobin; MCV: mean corpuscular volume; Fe: serum iron concentrations; YYIR1: Yo-Yo Intermittent Recovery Test Level 1; DSt: dribbling shuttle test; nbt: number of balls touching. * Significance at *p* < 0.05. ** Significance at *p* < 0.01.

**Table 2:** Prevalence of effective shots on goal accuracy tests among soccer players from EG and CG (µ and 95% CI for proportion).

Test	EG (Mean, 95% CI)	CG (Mean, 95%CI)
1 goal from 5 tries	0.23 (0.08; 0.47)	0.17 (0.04; 0.39)
2 goals from 5 tries	0.27 (0.10; 0.51)	0.20 (0.06; 0.43)
3 goals from 5 tries	0.30 (0.13; 0.54)	0.33 (0.15; 0.57)
4 goals from 5 tries	0.17 (0.04; 0.39)	0.23 (0.08; 0.47)
5 goals from 5 tries	0.03 (0.0003; 0.21)	0.07 (0.005; 0.26)

Note: EG: experimental group; CG: control group; CI: confidence interval. *p* = 0.8602.

**Table 3:** Anthropometric profile in soccer players from CG and EG (µ; 95%CI).

Parameters	EG (Mean, 95% CI)	CG (Mean, 95% CI)	*p*-Value	Cohen’s d
Anthropometrical parameters				
H (cm)	174.03 (172.64; 175.42)	175.27 (173.43; 177.16)	0.48	–
BM (kg)	67.84 (65.16; 70.52)	64.14 (61.84; 66.44)	0.04 *	0.55
BMI (kg/m^2^)	22.39 (21.39; 23.18)	21.99 (21.24; 22.74)	0.54	–
SH (cm)	85.99 (83.69; 88.29)	81.91 (79.52; 84.30)	0.01 *	0.64
LL (cm)	88.04 (86.14; 89.94)	93.69 (92.06; 95.33)	0.001 **	1.19
Diameters				
BACD (cm)	34.26 (33.41; 35.11)	32.33 (31.10; 33.53)	0.02 *	0.69
APCD (cm)	18.82 (18.20; 19.43)	17.84 (17.44; 18.26)	0.02 *	0.71
PBD (cm)	27.45 (26.91; 27.99)	26.08 (25.22; 29.94)	0.01 *	0.71
WD (cm)	6.06 (5.85; 6.22)	5.81 (5.68; 5.95)	0.01 *	0.53
KD (cm)	9.07 (8.81; 9.35)	8.55 (8.34; 8.76)	0.001 **	0.83
ED (cm)	8.86 (8.57; 9.17)	8.25 (7.99; (8.51)	0.001 **	0.82
AD (cm)	7.12 (7.01; 7.84)	6.84 (6.62; 7.07)	0.02 *	0.66
Circumference				
UAC (cm)	30.45 (29.50; 31.40)	32.28 (30.76; 33.80)	0.11	–
CC (cm)	35.03 (34.22; 35.85)	35.60 (34.96; 36.24)	0.26	–
FC(cm)	26.79 (26.19; 27.39)	26.36 (25.75; 26.98)	0.48	–
TC (cm)	56.10 (54.73; 57.47)	54.27 (52.80; 55.73)	0.10	–
Skinfold				
TS (cm)	0.65 (0.60; 0.71)	0.64 (0.58; 0.69)	0.81	–
FS (cm)	0.60 (0.53; 0.67)	0.55 (0.49; 0.61)	0.28	–
SS(cm)	0.81 (0.75; 0.87)	0.78 (0.72; 0.83)	0.38	–
MBS (cm)	0.94 (0.82; 1.06)	0.89 (0.80; 0.98)	0.64	–
SS (cm)	0.76 (0.69; 0.83)	0.68 (0.61; 0.76)	0.05 *	0.34
FTS (cm)	0.88 (0.74; 1.02)	0.74 (0.67; 0.81)	0.22	–
CBS (cm)	0.11 (0.10; 0.12)	0.10 (0.09; 0.10)	0.02 *	0.60
Somatotype profile				
Ectomorph	2.71 (2.35; 3.08)	3.52 (3.10; 3.95)	0.01 *	0.76
Mesomorph	5.98 (5.64; 6.33)	5.42 (5.08; 5.77)	0.02 *	0.61
Endomorph	2.18 (2.00; 2.37)	2.04 (1.84; 2.24)	0.19	–

Note: EG: experimental group; CG: control group; CI: confidence interval; H: height; BM: body mass; BMI: body mass index; SH: sitting height; LL: leg length; BACD: biacromial chest diameters; APCD: anterior-posterior chest diameters; PBD: pelvic biiliac diameter; WD: wrist diameter; KD: knee diameter; ED: elbow diameter; AD: ankle diameter; UAC: upper arm circumference; CC: calf circumference; FC: forearm circumference; TC: thing circumference; TS: triceps skinfold; FS: forearm skinfold; SS: subscapular skinfold; MBS: mid-belly skinfold; SS: suprailiac skinfold; FTS: front thigh skinfold and CBS: calf back skinfold; somatotype profile (See Figure 3) * Significance at *p* < 0.05. ** Significance at *p* < 0.01.

## Data Availability

Data are available for research purposes upon reasonable request to the corresponding author.
